# Uncovering the chemistry behind inducible morphological defences in the crustacean *Daphniamagna* via micro-Raman spectroscopy

**DOI:** 10.1038/s41598-020-79755-4

**Published:** 2020-12-29

**Authors:** Sven Ritschar, Vinay Kumar Bangalore Narayana, Max Rabus, Christian Laforsch

**Affiliations:** Department of Animal Ecology I, University of Bayreuth, Bayreuth, Germany

**Keywords:** Chemical biology, Ecology, Ecology, Limnology

## Abstract

The widespread distribution of Crustacea across every aquatic ecological niche on Earth is enabled due to their exoskeleton's versatile properties. Especially mineralization of the exoskeleton provides protection against diverse environmental threats. Thereby, the exoskeleton of some entomostracans is extremely phenotypically plastic, especially in response to predators. For instance, the freshwater zooplankton *Daphnia* forms conspicuous inducible morphological defenses, such as helmets, and can increase the stability of its exoskeleton, which renders them less vulnerable to predation. In this study, we reveal for the first time the chemical composition of the exoskeleton of *Daphnia* *magna,* using Raman spectroscopy*,* to be composed of α-chitin and proteins with embedded amorphous calcium carbonate (ACC). Furthermore, we reveal the exoskeleton's chemical changes associated with inducible defense mechanisms in the form of more substantial mineralization, which is probably correlated with enhanced carapace stability. We, therefore, highlight the importance of calcium-biominerals for inducible morphological defenses in *Daphnia*.

## Introduction

The mineralized exoskeleton, or cuticle of crustaceans, provides an effective defense against abiotic and biotic threats^1,2^. In malacostracans, the exoskeleton consists of an organic matrix with embedded biominerals. For instance, the exoskeletons of the American lobster (*Homarus* *americanus)* and the brown crab (*Cancer* *pagurus*) consist mainly of crystalline magnesium calcite and amorphous calcium phosphate (ACP) embedded in an organic matrix composed of α-chitin^3^. The current research focus is, in particular, aimed at studying the mechanisms associated with calcification of the exoskeleton, e.g. investigating the proteins involved in this process and associated regulatory mechanisms^4–6^. Moreover, the various structural properties and biomineralization of exoskeletons have been investigated in many crustacean species, like blue crabs (*Callinectes* *sapidus*)^2,7^, sheep crabs (*Loxorhynchus* *grandis*)^8^, grapsid crabs (*Metopograpsus* *fontinalis*)^9^ and freshwater crayfish (*Cherax* *quadricarinatus*)^10^. While this research focuses primarily on malacostracans, investigations of the cuticle of entomostracans are rare, and only a few studies provide information on its composition, while predominantly using macroscopic techniques^11^. In some entomostracans, like the subgroup of the Cladocera, the exoskeleton is divided into a so-called carapace, which is the part of the exoskeleton that’s encaging the main part of the body, and a helmet which is a head shield^12^.

Thereby, the carapace itself is well understood as a natural barrier or defense against abiotic or biotic stressors, such as predators. Since predation is a strong selective force, both constitutive and inducible antipredator defensive traits have evolved^13^. Inducible defenses, representing a form of phenotypic plasticity, are an appropriate mechanism to cope with the variable impact of a frequently changing predator spectrum. It allows a specific defense in a prey organism to manifest itself only if a reliable cue for a pending attack is given^14^. This means the prey organisms can reduce costs related to the formation or maintenance of defense mechanisms, while the predation risk is low^15^. Besides providing individual benefits for the prey, their ecological relevance can be seen in structuring food-web interactions as they dampen predator–prey oscillations and promote stability in multi-trophic communities^16^. Hence, phenotypic plasticity in defensive traits is found in a variety of taxa spanning from protists^17^ to vertebrates^18^. Among the most striking examples of inducible defenses are morphological defenses displayed by entomostracans of the genus *Daphnia*. In response to several invertebrate predators, they develop adaptive morphological changes of their exoskeleton, such as the generation of crests, helmets and spines^19–21^. Although these conspicuous induced traits have been proven to reduce predator caused mortality, the exact defensive mechanisms remain unresolved.

It has been previously described that daphnids are able to increase the stability of their exoskeleton in response to predation. For instance, *D.* *cucullata* and *D.* *pulex* show 300-fold increased stability when threatened by the phantom midge *Chaoborus* *flavicans*^22^. Similar to this study, Rabus et al.^23^ characterized the fortification of the carapace of *D.* *magna* as an inducible defense mechanism against *Triops* predation. The very distinct fortification of *Daphnia*'s carapace, which involves its restructuring and reinforcing observed in these studies, implies that prominent chemical modifications may accompany these changes. However, there is still a lack of knowledge of these potential chemical modifications underlying the carapace's fortification.

Previous studies that focused on proteomic and transcriptomic investigations of inducible defenses in *Daphnia* indicate that calcium associated proteins are more upregulated when inducible defenses are expressed^24,25^. Therefore, we hypothesize that the composition and abundance of inorganic constituents such as calcium-based biominerals and organic constituents such as α-chitin within the carapace may play a vital role in fortifying the carapace. To address this, we used micro-Raman spectroscopy, a vibrational spectroscopy technique that has been demonstrated widely for the non-invasive monitoring of biomolecules in different native systems, ranging from a single cell to complex tissues, in the last two decades^26–29^. Micro-Raman spectroscopy allows the acquisition of a molecular fingerprint from biological samples at a high spatial resolution of ~ 1 µm consisting of signatures from biomolecules such as nucleic acids, carbohydrates, lipids, proteins and also inorganic constituents^26^. This gives the technique a huge advantage in investigating the chemistry of a sample with or without the influence of a physical or a physiological perturbation factor. Herein, we apply this approach to give a detailed description of the chemical composition of the carapace of *D.* *magna* for the first time. We further investigate the chemical changes occurring in the carapace of the Crustacean *Daphnia* concerning the expression of inducible morphological defenses triggered on exposure to the predator *T.* *cancriformis*.

## Results

### Characterization of the chemistry of the components constituting the carapace of *D. magna*

**Figure 1:**
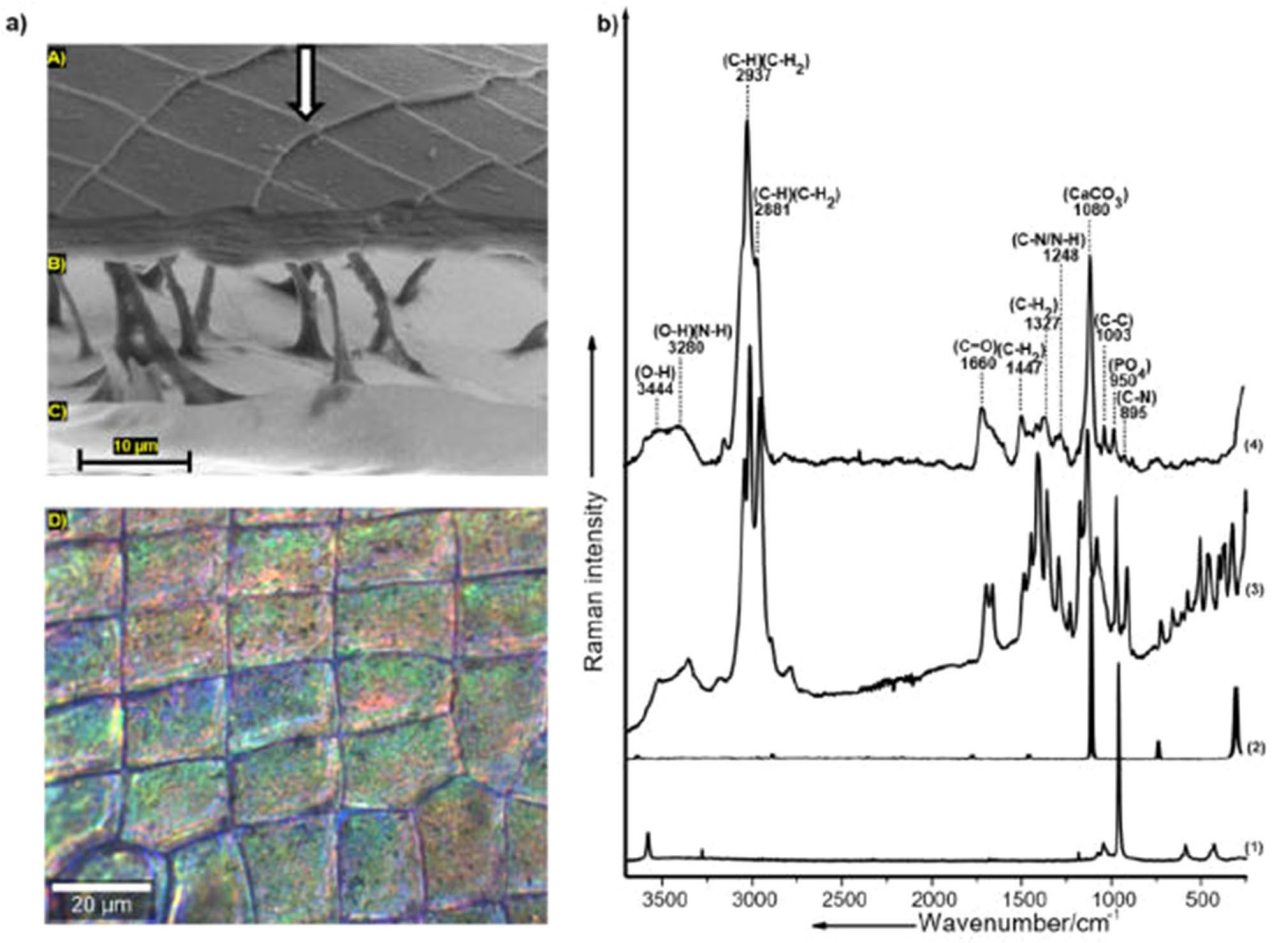
Characterization of the chemistry of the distal integument of the carapace of *D.* *magna* via micro-Raman-spectroscopic-analysis; **(a)** SEM image of the carapace of *D.* *magna* highlighting the features of the carapace (A) distal integument (B) hemolymphatic chamber (C) proximal integument and (D) exemplary bright-field microscopic image representing the surface of the distal integument of the carapace used for the acquisition of Raman spectra (white arrow in the SEM image indicates the focal plane of the bright field microscopic image **(b)** mean Raman spectrum (calculated from n = 200 spectra acquired from 8 replicates (carapace samples) per treatment) of the distal integument of the carapace overlapped over spectra of the reference substances; (1) Hydroxyapatite reference, (2) Calcite reference, (3) α-Chitin reference and (4) Distal integument of the carapace.

The carapace of *D.* *magna*, in general, consists of two opposing layers called proximal (inner layer) and distal (outer layer) integument^11,30^ (Fig. 1a). The distal integument acts as the main barrier to the outer environment, and therefore, our analysis was focused on investigating the chemistry associated with the distal integument of the carapace. Micro-Raman spectroscopic analysis revealed that the distal integument of *D.* *magna* consists of key molecules, mainly α-chitin and proteins constituting the organic components and amorphous calcium carbonate (ACC) and phosphates making up the inorganic counterparts. The mean Raman spectrum acquired from the carapace samples can be interpreted as a combination of Raman bands arising from both the organic and inorganic components (Table 1, Fig. 1b). The Raman bands at 895, 950, 1003 and 1080 cm^−1^ are associated with symmetric stretching vibrations of the (C–N) bond (contribution from α-chitin and proteins), phosphates (PO_4_^3^) (phosphorylated proteins and may be ACP), (C–C) ring breathing mode of phenylalanine (proteins) and carbonates from calcium-based biomineral (CaCO_3_), respectively^30,32^. The Raman bands at 1248, 1327 and 1447 cm^−1^ are associated with bending vibrations of (C–H) and (C–H_2_) bonds (contribution from α-chitin and proteins) whereas the Raman band at 1660 cm^−1^ is associated with stretching vibrations of amide (C=O) bonds (contribution from α-chitin and proteins mainly)^29,31^. The Raman bands at 2881, 2937, 3280, and 3444 cm^−1^ are associated with stretching vibrations of (C–H), (C–H_2_), (N–H) bonds (contribution from α-chitin and proteins) and (O–H) bonds (ACC and α-chitin), respectively^29,31^. The presence of specific above mentioned organic and inorganic components could be interpreted based on the comparison of the mean Raman spectrum of the carapace samples with the reference spectrum acquired from α-chitin, calcite (crystalline CaCO_3_) and hydroxyapatite (crystalline Ca_5_(PO_4_)^3^(OH)) respectively (Table 1, Fig. 1b). The presence of broad Raman bands with regards to calcium carbonate stretching vibration (1080 cm^−1^) coupled with the presence of (O–H) bonds representing water molecules (3280, 3444 cm^−1^) indicates that the biomineral phase in the carapace of *D.* *magna* is in the amorphous form (Fig. 1b (4)). This is confirmed by comparing the carapace samples Raman spectrum with the spectrum of calcite, which is a crystalline phase of the biomineral indicating sharp Raman bands and the absence of signatures corresponding to water molecules (Fig. 1b (2, 4)).

**Table 1:** Peak assignments corresponding to the chemical composition of the carapace of *D.* *magna*.

Peak (cm^−^^1^)	Assignments
895 950	Symmetric stretching vibrations of the (C–N) bond (α-chitin and proteins) Symmetric stretching vibrations of (PO_4_^3^) phosphates (phosphorylated proteins and may be ACP)
1003	Symmetric ring breathing mode of (C–C) phenylalanine (proteins)
1080	Symmetric stretching vibrations of (C–O_3_) carbonates (amorphous calcium carbonate (ACC))
1248	Bending and wagging vibrations of (C–H) and (C–H_2_) bonds (α-chitin and proteins)
1327	Bending and wagging vibrations of (C–H) and (C–H_2_) bonds (α-chitin and proteins)
1447	Bending and wagging vibrations of (C–H) and (C–H_2_) bonds (α-chitin and proteins)
1660	Stretching vibrations of amide (C=O) bonds (α-chitin and proteins)
2881	Symmetric and asymmetric stretching vibrations of (C–H) and (C–H_2_) bonds (α-chitin and proteins)
2937	Symmetric and asymmetric stretching vibrations of (C–H), (C–H_2_) and (N–H) bonds
3280	(α-chitin and proteins) Stretching vibrations of (O–H) and (N–H) bonds (ACC and α-chitin)
3444	Stretching vibrations of (O–H) bonds (ACC)

### Arrangement of the components constituting the carapace of *D. magna*

**Figure 2:**
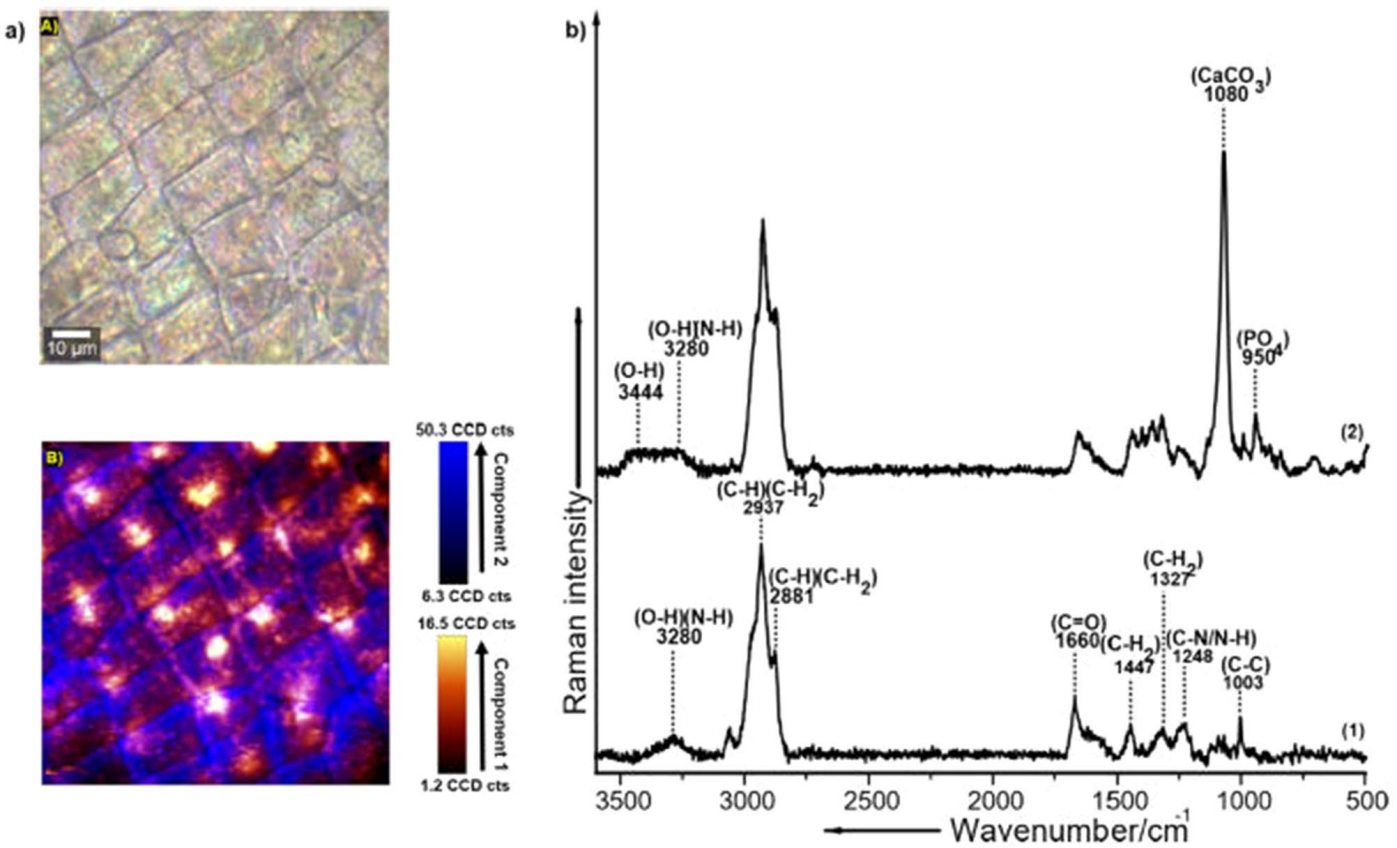
Distribution of organic and inorganic components within the carapace of *D.* *magna* visualized via micro-Raman spectroscopic imaging; **(a)** (A) Bright-field microscopic image representing the distal integument of the carapace and (B) corresponding false colored Raman image acquired from an exemplary replicate sample indicating intensity-based differential distribution of the demixed spectral components color coded in orange (component 1) and blue (component 2), **(b)** Demixed Raman spectrum representing the organic and inorganic components of the carapace: (1) Component 1, representing spectral signatures corresponding to organic components (α-chitin and proteins) and (2) Component 2, representing spectral signatures comprising of both organic and inorganic components (ACC and phosphates). The color scale represents the relative intensity distribution (standard deviation) of component 1 and component 2 within the Raman image, respectively. The spectra of component 1 and component 2 are representing the respective mean spectrum calculated from n = 22,500 spectra constituting the Raman image analyzed via TCA analysis coupled with spectral demixing. The Raman image has been acquired from one *D.* *magna* carapace sample (n = 1).

The arrangement of organic and inorganic components within the distal integument of the carapace was then further investigated. Micro-Raman imaging visualized that the carapace of *D.* *magna* comprises of all the organic and inorganic components mentioned in the section above, which showed an intricate pattern of differential distribution across the surface (Fig. 2). In the Raman image, intensity-based differential distribution of these components can be observed throughout the carapace with the orange intensity scale indicative of spectral component 1 and the blue intensity scale of spectral component 2, respectively (Fig. 2a). The more intense color indicates a higher concentration of the respective chemical components and vice versa. The Raman bands in demixed spectral component 1 (Fig. 2b (1), Table 1) seen at 1003, 1248, 1327, 1447, 1660, 2881, 2937, and 3280 cm^−1^ can be exclusively assigned to the organic components (contribution from α-chitin and proteins)^29^. Whereas, the demixed spectral component 2 (Fig. 2b (2), Table 1)) comprised of less intense Raman bands which arise from the organic components mentioned above, as well as prominent bands at 950, 1080, 3280 and 3444 cm^−1^ which can be assigned to phosphates and ACC respectively ^29,31^.

### Detection of key components of the carapace of *D. magna* in association with the expression of inducible morphological defenses

#### Analysis of morphological parameters

**Figure 3:**
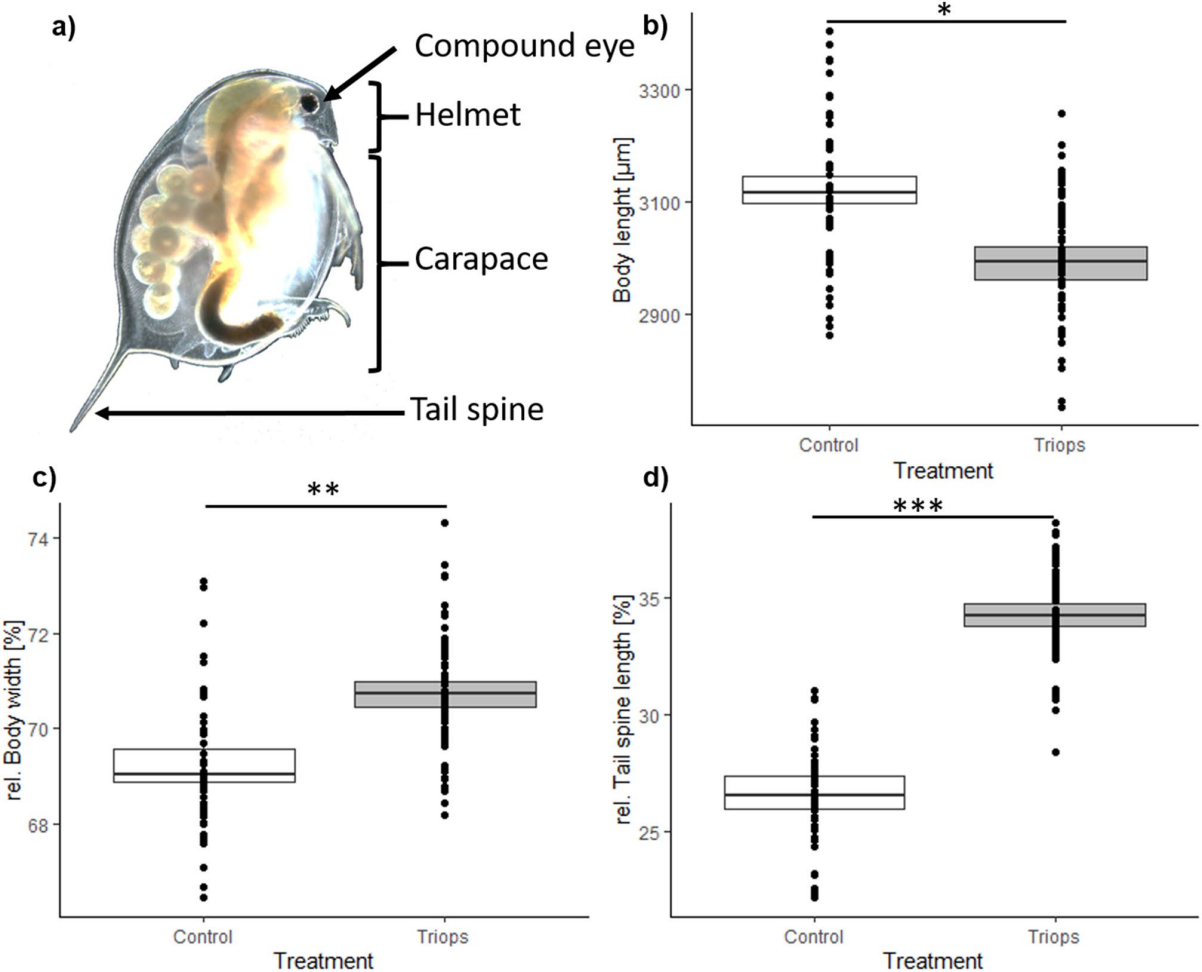
Comparison of *Triops*-exposed and non-exposed *D.* *magna*. **(a)** Bright-field microscopic image of an individual *D.* *magna*. Comparison of **(b)** body length, **(c)** relative body width and d) relative tail spine length of *Triops*-exposed (*Triops*) and non-exposed (Control) *D.* *magna*. The black dots in **(b–d)** display the measurement of the respective body parameters of every animal used in the induction experiment, which is a total of 60 animals per treatment (Control vs *Triops*), separated in 6 replicates (1 replicate with 10 individual *D.* *magna*). The data is displayed as **(b)** the raw data (µm) and for **(c,d)** the relative values (%). Asterisks indicate statistically significance: * = *P* < 0.05, ** = *P* < 0.01 and *** = *P* < 0.001.

Firstly, the expression of *Triops*-induced morphological defenses was evaluated by analyzing the morphological parameters, which have been previously proven to function as successful defensive traits that increase the survival rate of *D.* *magna*^13,32^ (Fig. 3): In this context, our results showed a significant increase in relative body width (t-test; t(10) = − 4.575; *P* = 0.001) and tail spine length (t-test; t(10) = − 8.952; *P* < 0.001) in *D.* *magna* exposed to the predator which is in accordance with previous publications^32,33^, except for body length which was shorter compared to non-exposed ones (t-test; t(10) = 2.737, *P* = 0.021).

#### Detection of key chemical components influencing inducible morphological defences

**Figure 4:**
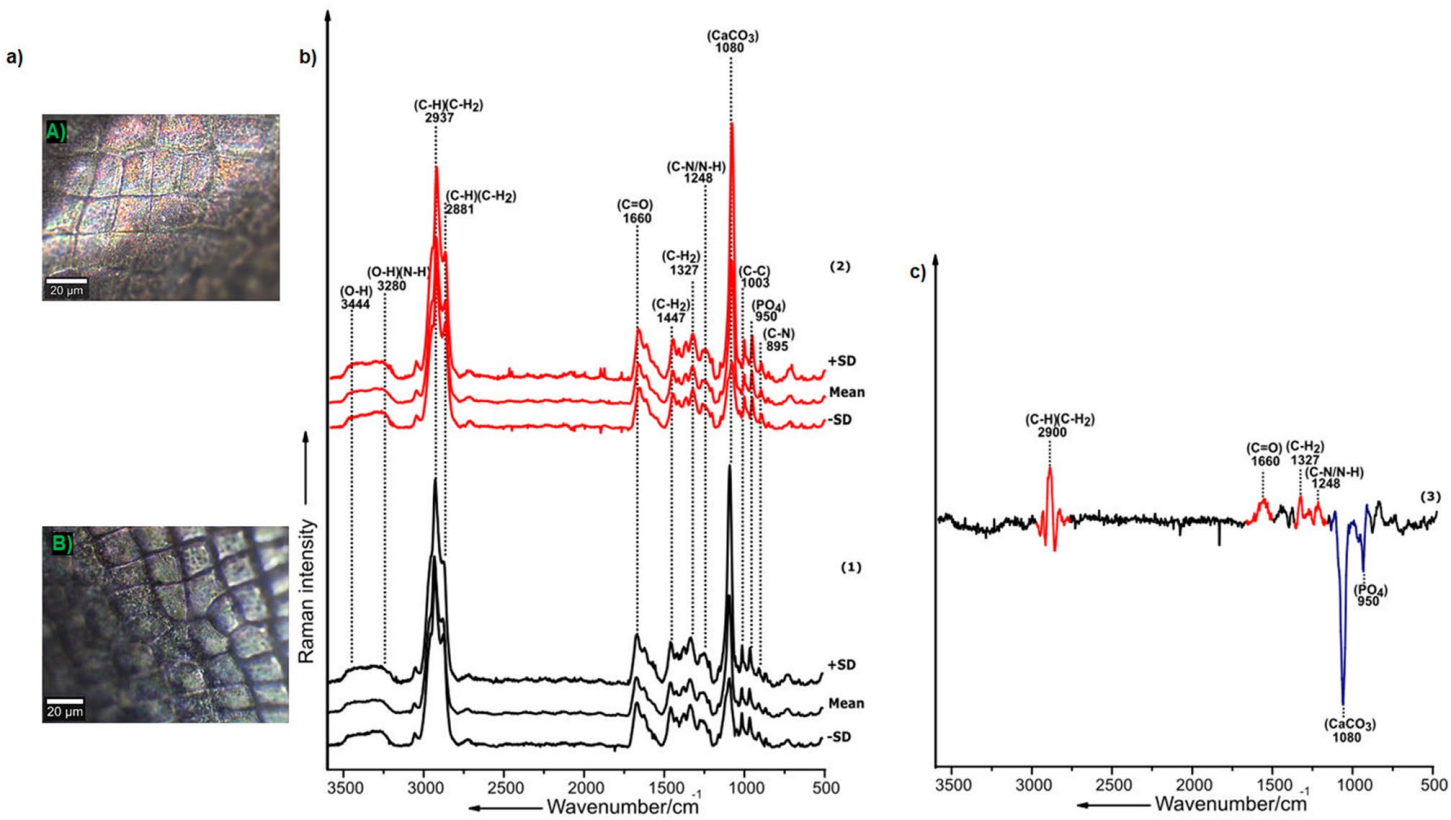
Detection of key chemical components of the carapace of *D.* *magna* in association with inducible defenses; **(a)** exemplary Bright-field microscopic images corresponding to the distal integument of the carapace (A) Carapace of control *D.* *magna* (B) Carapace of *Triops*-exposed *D.* *magna,* **(b)** mean Raman spectrum ± 1 standard deviations (SD) of the respective carapace samples (calculated from n = 200 Raman spectra, 25 per replicate acquired from 8 replicates (carapace samples) per group i.e. control and *Triops-*exposed *D.* *magna*); (1) Carapace of individuals of the control; (2) Carapace of *Triops*-exposed *D.* *magna;* c) Difference spectrum (calculated from n = 400 Raman spectra acquired from the 8 respective carapace samples of the two groups (Control and *Triops*-exposed *D.* *magna*) highlighting the major spectral differences between the carapaces of non-exposed (red colored regions) and *Triops*-exposed *D.* *magna* (blue colored regions).

The results of the micro-Raman analysis revealed that *Triops*-exposed *D.* *magna* contain a relatively higher concentration of ACC and phosphates in their carapace compared to the non-exposed *D.* *magna* (Fig. 4). This increase in the biomineral content constituting ACC and phosphates might be associated with contributing better mechanical properties to the distal integument^34^. The profiles of the Raman spectra obtained from both morphotypes summarized as mean spectrum coupled with ± 1 standard deviation (SD) indicate the presence of organic and inorganic component signatures, as described in the section above (Fig. 4b (1,2)). As can be seen from the difference Raman spectrum, the major differences between the carapace samples of non-exposed and *Triops*-exposed *D.* *magna* are observed in the Raman bands at 950, 1080, 1248, 1327, 1660 and 2900 cm^−1^ respectively (Fig. 4c). The Raman bands corresponding to phosphates (contribution from phosphorylated proteins and maybe ACP) and ACC (950 and 1080 cm^−1^ blue-colored regions)^29^ are significantly more intense in the spectrum of *Triops*-induced carapace samples in comparison to the control samples, which indicates a higher concentration of these components (Fig. 4c). In contrast, the Raman bands at 1248, 1327, 1660, and 2900 cm^−1^ (C–H, C–H_2_ stretching vibration-red colored regions)^29^ show a higher intensity in the carapace samples of non-exposed daphnids, highlighting the relatively higher concentration of organic components constituting mainly α-chitin and proteins (Fig. 4c). However, it has to be mentioned that a higher concentration of ACC may overlay the organic components within the carapace, which may be a reason for the relatively reduced intensities of the Raman bands corresponding to organic components observed in the spectrum of the *Triops*-exposed *D.* *magna*.

**Figure 5:**
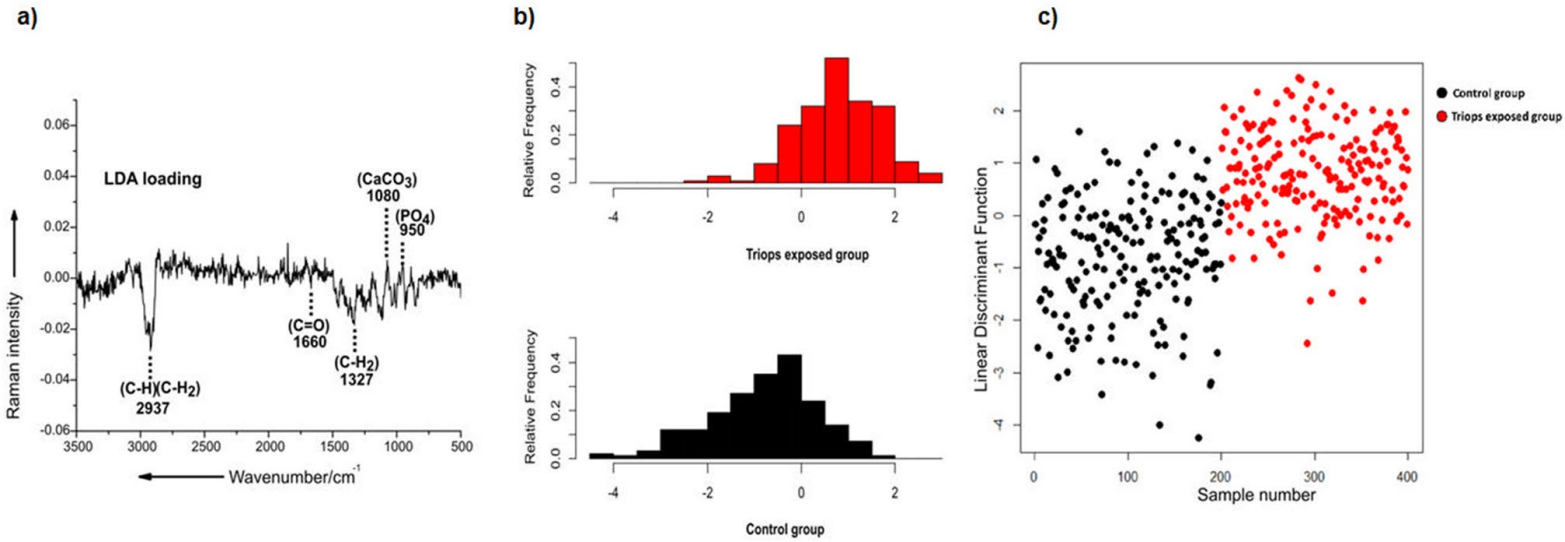
PCA-LDA analysis of the Raman spectra acquired from the carapace samples of control and *Triops*-exposed *D.* *magna*; **(a)** loading of the PCA-LDA model for the differentiation of carapace samples of control and *Triops-*exposed *D.* *magna*; **(b)** PCA-LDA histogram plot, the horizontal axis describes the linear discriminant function values and the vertical axis the relative frequencies of each class. The histogram coded in black represents the control group and that coded in red represents the *Triops*-exposed group. The data analyzed was obtained from 8 replicates (carapace samples) per group constituting 25 spectra per replicate, meaning 200 spectra per treatment and a total of 400 spectra acquired across the two groups; **(c)** PCA-LDA score plot showing the variation between the two groups of samples, the black dots represent all the spectra of the control group (n = 200) and the red dots represent all the spectra of the *Triops*-exposed group (n = 200).

Furthermore, a classification model was developed using principal component analysis coupled with linear discriminant analysis (PCA-LDA) in an effort to distinguish between the carapace samples of *D.* *magna* from the control group and the *D.* *magna* from the *Triops-*exposed group. The PCA-LDA histogram plot and the score plots show the distribution of Raman data across the discriminant function values and indicate the possibility to separate the two groups of samples i.e. the carapace samples of the control group from the predator-exposed group with an accuracy of 76% (Fig. 5b,c). Although, there is a certain amount of overlap between the two groups as seen clearly in the PCA-LDA histogram plot, the data of the control group is mostly distributed towards the negative direction and the *Triops*-exposed group is distributed towards the positive direction of the discriminant function (Fig. 5b). Furthermore, in Fig. 5c, it is shown that the Raman spectra of the carapace samples of *D.* *magna* from the control group are largely distributed at the negative LD score values, whereas those of the individuals from the *Triops-*exposed group are mostly distributed at the positive LD score values. The respective LDA loading (Fig. 5a) visualizes the spectral differences used by the PCA-LDA model for distinguishing between the carapace samples of *D.* *magna* from the control and the *Triops-*exposed group. The correlation of LDA score values with the LDA loading (Fig. 5a) supports the presence of a relative high abundance of phosphates and ACC (950 and 1080 cm^−1^) in the carapace samples of the *Triops-*exposed *D.* *magna* (Fig. 5). In contrast, the carapace samples of the control group visualize a negative LD coefficient around 1327, 1660 and 2937 cm^−1^ which can be attributed to a higher abundance of organic components constituting mainly α-chitin and proteins. The level of abundance and distribution of all these organic and inorganic components can be further exemplarily visualized across the carapace via high-resolution large scale Raman imaging analysis of an exemplary carapace sample of the two groups.

#### Mapping the distribution of ACC and phosphates within the carapace of predator-exposed *D. magna*

**Figure 6:**
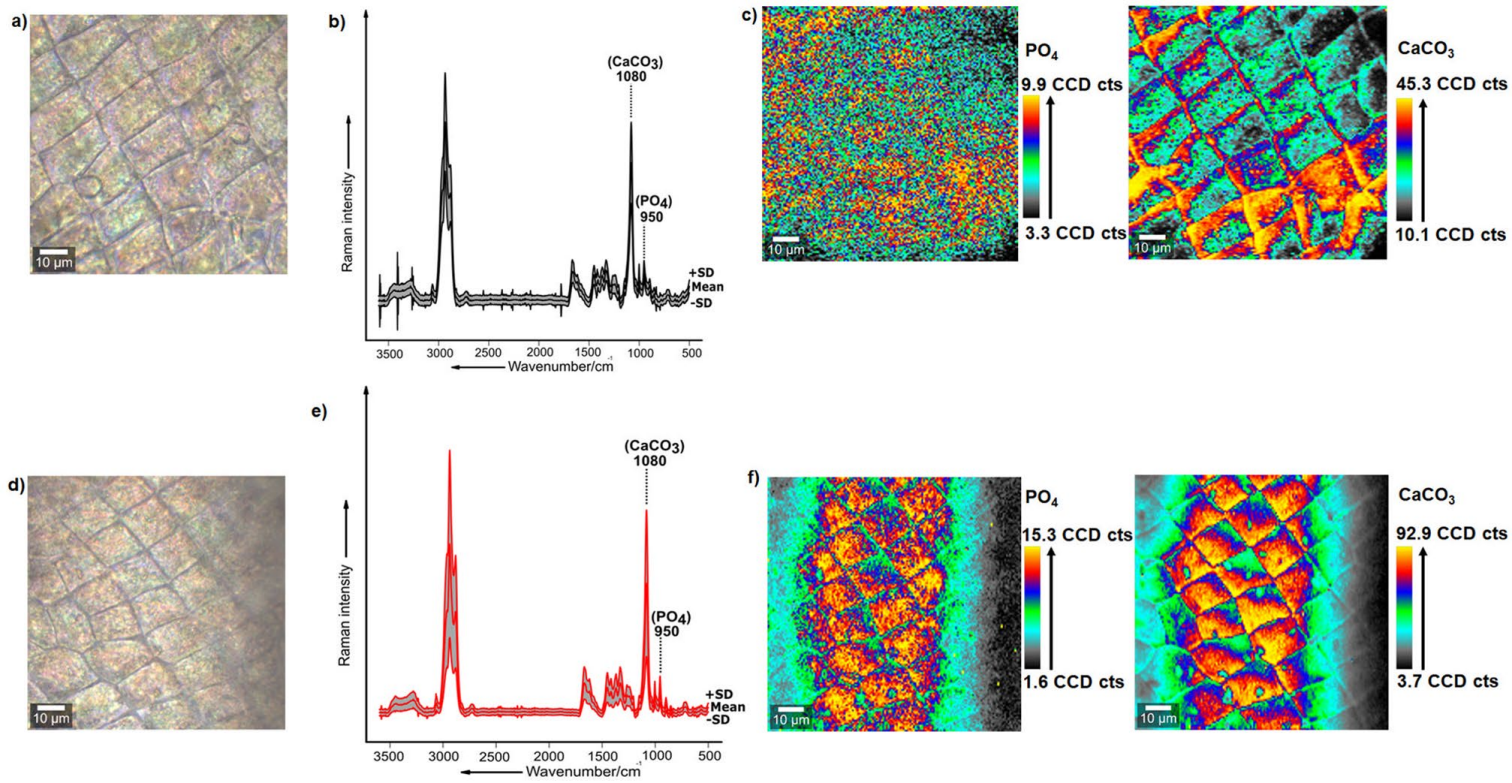
Distribution of ACC and phosphates within an exemplary control carapace sample and *Triops*-exposed carapace sample of *D.* *magna* monitored via micro-Raman spectroscopic imaging. Upper panel: **(a)** Bright-field microscopic image of the surface of the control carapace sample, **(b)** mean Raman spectrum ± 1 standard deviations (SD) of the respective carapace (calculated from n = 22,500 Raman spectra) with regions highlighting the Raman bands corresponding to phosphates (950 cm^−1^) and ACC (1080 cm-^1^) respectively, **(c)** False colored Raman images acquired from a control carapace sample (n = 1) indicating the differential intensity distribution of the Raman bands corresponding to phosphates (left) and ACC (right) respectively. Lower panel: **(d)** Bright-field microscopic image of the surface of the *Triops-*exposed carapace sample, **(e)** mean Raman spectrum ± 1 standard deviations (SD) of the respective carapace (calculated from n = 22,500 Raman spectra) with regions highlighting the Raman bands corresponding to phosphates (950 cm^−1^) and ACC (1080 cm^−1^), respectively, **(f)** false colored Raman images acquired from a *Triops*-exposed carapace sample (n = 1) indicating the differential intensity distribution of the Raman bands corresponding to phosphates (left) and ACC (right), respectively. CCD counts (CCD cts.) on the color scale represents relative intensity variation of Raman signatures corresponding to phosphates and ACC within the Raman image.

The distribution and relative concentration of the components, ACC and phosphates, which were the main components influencing changes in the chemistry of the carapace of *D.* *magna* as a response to predation by *T.* *cancriformis*, was then further investigated using micro-Raman spectroscopic imaging analysis exemplarily on two replicates per treatment. The Raman images were acquired to provide support that the pattern of results obtained in single point to point measurements are observed in large areas spanning the respective carapaces. Firstly, bright-field images of the respective non-exposed and *Triops*-exposed *D.* *magna* carapace sample were visualized (Fig. 6a,d). Secondly, the data from the Raman images acquired were summarized as mean spectrum coupled with ± 1 standard deviation (SD) (Fig. 6b,e). The Raman images depict the differential distribution and relative concentration of ACC and phosphates within the carapace sample of the non-exposed (Fig. 6c) and *Triops*-exposed *D.* *magna* (Fig. 6f). Herein, ACC is mapped based on the standard deviation of integrated normalized Raman intensities corresponding to the Raman band at 1080 cm^−1^, and phosphates are mapped based on the intensities of the Raman band at 950 cm^−1^ (Fig. 6c,f). It is observed from the Raman images that the relative concentration of ACC and phosphates are significantly higher in the carapace sample of *Triops*-exposed *D.* *magna* (Fig. 6f). It becomes apparent that ACC and phosphates are distributed across the carapace's entire area in *Triops*-exposed daphnids, depicting regions that are highly concentrated with ACC and phosphates. In contrast, Raman images of the carapace samples of control animals depicted lower concentrations of ACC and phosphates (Fig. 6c). These results were obtained from a single biological replicate of samples, one individual for each of the control group and *Triops-*exposed group. The Raman imaging results from a second replicate of samples which followed same patterns have been included in the SI (Figure S1).

## Discussion

### Nature and arrangement of the components constituting the carapace of *D. magna*

**Figure 7:**
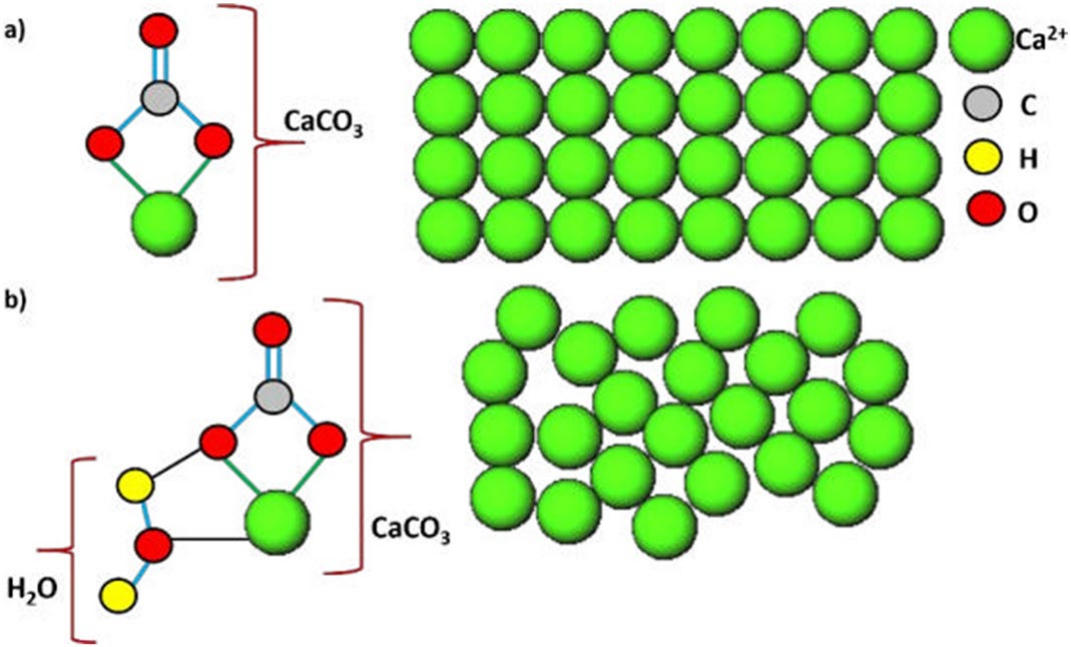
Arrangement of atoms in the amorphous phase vs crystalline phase of calcium carbonate (CaCO_3_); **(a)** crystalline calcium carbonate (calcite), **(b)** Amorphous monohydrated ACC; spheres colored in green represent calcium ions (Ca^2+^), grey represent carbon atoms (C), yellow represents hydrogen atoms (H) and red represents oxygen atoms (O) respectively.

The arrangement of the components constituting the carapace of *D.* *magna* may be interpreted as a type in which the inorganic components (ACC and phosphates) are embedded into a matrix comprising the organic components (α-chitin and proteins). While calcium-biominerals such as ACC have been previously found in the exoskeleton of malacostracan crustaceans, e.g. lobster*s*^3,35^, crabs^3^ and crayfish^36^*,* it has not been shown to be present in entomostracan Cladocera. Therefore, our study is the first to reveal that the entomostracan, *D.* *magna*, utilize ACC as a biomineral in their carapace. An essential difference in the composition of the exoskeleton between *Daphnia* and malacostracans is the arrangement of calcium carbonate in an amorphous form instead of an additional crystalline form of calcium carbonate (calcite) that is observed in Malacostraca^35,37,38^. ACC is the hydrated polymorph of the calcium carbonate mineral and different levels of dehydration lead to its transformation into crystalline calcium carbonate (e.g. calcite), which can involve various processes such as nucleation^39^. Calcite possesses an ordered arrangement, whereas ACC has a disordered arrangement (Fig. 7)^40^. This somewhat unstable arrangement is thought to have the advantage to be much more flexible and therefore, might be easier fitted for specific functions^41^. For *D.* *magna* this could involve processes like storage, transportation, or mineralization of the carapace.

Even though no crystalline form of calcium carbonate biominerals could be found in the integument of *D.* *magna* in our study, there is evidence that this organism can also use calcium-based biominerals in a crystalline form. For instance, Kawasaki et al.^42^ detected the presence of the crystalline calcium phosphate (apatite) in the ephippia of *D.* *magna.* Herein, the ephippium, which encages two resting eggs of *Daphnia*, consists of a thick layer of calcium phosphate enclosing the embryos, which is then surrounded by a layer of chitin and pigments like melanin^43^. The results observed from our analysis showed the presence of phosphates but due to overlapping of Raman bands corresponding to phosphates from both organic and inorganic components. It was not possible to infer if they might be in sole coordination with calcium, which might then be assigned as amorphous calcium phosphates (ACP) or part of phosphorylated proteins representing the organic components of the carapace.

However, phosphates are known to play a vital role in the stabilization of ACC since proteins that have been described to stabilize ACC in malacostracan species are phosphorylated^6,10^. Interestingly, the moderately intense Raman band observed at 950 cm^−1^ representing stretching vibrations of phosphates in demixed spectral component 2 compared to demixed spectral component 1 may suggest the presence of ACP in addition to phosphorylated proteins. Furthermore, the differentiation between ACC and ACP is quite difficult due to the occurrence of a mixed ACC/ACP-phase (ACCP)^44^, in which phosphates are again said to be key for the stabilization of ACC ^10,45^. Nevertheless, the presence of these amorphous biomineral phases is known to provide a flexible tool for organisms that can be modified for specific functions^41^, like storage. It exists as a precursor phase of crystalline calcium carbonate and still provides enhanced stability^45^. Therefore, these biominerals may be significantly advantageous for the phenotypically plastic genus *Daphnia* and likely changes in parameters such as their concentration, phase transition, or pattern of embedding into the organic matrix may play an essential role in the expression of inducible morphological defenses. Our results are therefore in concordance with previous reports about, and add a detailed description to, the vital role of elemental calcium and phosphorous for *Daphnias*’ carapace formation ^46^.

An additional study that needs to be mentioned in this context is of Wærvågen et al.^47^. Here, the authors reported, that the calcium concentration influences the distribution of *Daphnia* species and is correlated with body size, allowing a reflection on their individual demand. The shown correlation of calcium concentration with body size ^47^ is exciting, given the fact that many *Daphnia* species are known to vary their body sizes as a results of phenotypic plastic responses to environmental stressors like predation ^20,21^. Therefore, it may be important to analyse parameters like calcium concentration of *Daphnias*’ carapace in response to varying biotic and abiotic stressors.

### Key carapace components influencing the expression of inducible morphological defenses in *D. magna*

The verification of the expression of inducible defenses in *D.* *magna* is indicated by changes in the parameters body length, body width, and tail spine length which were proven to significantly reduce mortality when attacked by *T.* *cancriformis* ^33^. The only exception was body length, which tended to be smaller in defended animals compared to control animals. While these contrasting results may be explained by clonal or experimental procedure related variation, the observed change in morphology indicates that *D.* *magna* responded with the restructuring of its morphological features, i.e. body width and tail spine length, to the presence of *T.* *cancriformis*. These morphological changes in *D.* *magna* are coupled with a simultaneous increase in thickness and rigidity of the distal integument ^23^. Therefore, it may be envisioned that these morphological changes may be coupled with key changes in the carapace's chemistry.

The main chemical difference accompanying the morphological changes was the stronger mineralization of the carapace, in the form of increased ACC content in the carapace samples of *Triops*-exposed *D.* *magna* compared to the carapace samples of its unexposed counterpart. Nikolov et al.^48^ have shown that the stability of the cuticle of Lobsters, measured as Young’s modulus, improves with the increasing calcium content. Therefore, our results showing a higher ACC concentration may explain the predator-induced increase in the cuticle's rigidity, as shown in some *Daphnia* species^22,23^. For instance, the five-fold increase in cuticle rigidity in *Triops*-exposed *D.* *magna*^23^ may be influenced by a higher concentration of ACC incorporated into the cuticle. Furthermore, the Raman bands corresponding to phosphates, which also show increased intensities in the carapace samples of *Triops*-exposed *D.* *magna*, may be linked to biominerals such as ACP and phosphorylated proteins. This means that the phosphates (ACP and phosphorylated proteins together) are either involved in the stabilization of ACC, which ultimately influences the overall chemical composition of the exoskeleton in a direction that benefits the organism. Alternatively, the phosphates may be interacting with ACC as a means of a dual mineralization strategy (coexistence of ACC and ACP) for the better enforcement of the carapace. The latter would imply that the carapace consists of a mixed phase of ACC and ACP, which is discussed to play a role in stabilizing the biominerals in exoskeletons of crustaceans^35^. For representatives of the Malacostraca, it has also been previously reported that an increase in the ACP: ACC ratio can be associated with increased mechanical properties such as hardness and elastic modulus^49^.

Even though the degree and form of mineralization might be the major factor for carapace stability, it is essential to mention here that it is probably not the increase in mineralization alone that underlies the carapace's fortification and enforcement in *D.* *magna*. The carapace is a composite in which its mineral components are in specific coordination with α-chitin and proteins making up the organic matrix. The coordination of the components is crucial for the macroscopic appearance of the exoskeleton and enables crustaceans to organize their exoskeleton ^50^. Hence, it may contribute to the formation of a tough mechanically graded structure in which a strongly mineralized layer is mounted over a lesser mineralized organic matrix as a softer substrate, which will absorb mechanical stress and deflect potentially dangerous cracks on the exoskeleton ^51^.

Therefore, we provide a detailed chemical analysis of the changes related to inducible defenses to the previously described thickening of the distal integument^30^. In their studies, Kruppert et al.^30,52^, give a detailed analysis and background for structural changes within the distal integument of *Daphnia*, finding it more laminated and thicker, concluding that structural alterations are responsible for an increased carapace stiffness in predator-exposed individuals. To these findings, we add the knowledge, that processes of restructuring might coincide with a chemical rearrangement and boost of the chemical components involved in carapace formation in *Daphnia,* depicting ACC and phosphates to be key players involved.

### Significance of ACC and phosphates in the fortification of the carapace of predator-exposed *D. magna*

The results from Raman images of the distal integument indicated a differential distribution and increase in the relative concentration of ACC and phosphates in the *T.* *cancriformis-exposed* *D.* *magna* carapace samples compared to the non-exposed carapace samples. These key chemical modifications constituting ACC and phosphates might occur in synchronization with the thickening of the distal integument^30^ as a defensive response against predators. This is in concordance to a previous study that showed an increase in thickness of the distal integument as an inducible defensive response in *D.* *magna,* which might contribute to the overall stability of the carapace^23^. Hence, it may be established from our results that the morphological alterations such as thickening of distal integument, which are of importance in inducible defense responses in *D.* *magna,* are coupled with significant alterations in the overall chemistry of the distal integument.

Our results highlight the importance of calcium biominerals for the freshwater crustacean *D.* *magna*. It may be deduced that an increase in calcium biomineral and phosphate concentration may be a crucial chemical change occurring in association with the fortification of the cuticle in *D.* *magna*. Other studies have previously focused on the role of Calcium for daphnids, concluding that a reduction of calcium impacts *Daphnias*’ population growth ^53^ and also leads to a disabled functionality of its inducible defenses ^54^. Riessen et al. ^54^ have already discussed, that the strengthening of the carapace of *Daphnia* might require the availability of calcium and the ongoing calcium decline in *Daphnias*’ freshwater habitats impacts the functionality of its inducible defenses. In addition, the works of Tan and Wang^55^, Wærvågen et al.^47^ and Hessen and Rukke^46^ describe and highlight the importance of both calcium and phosphates for the freshwater crustacean *Daphnia*. In detail, the authors initiate the discussion, that *Daphnia* has a limited control of its calcium influx and efflux ^55^ and that moulting pose a constant drain of calcium for these organisms ^46^. These findings hint at a potential and immense cost of inducible morphological defenses since a higher Ca-biomineral content within the carapace of *Daphnia* would also imply a higher loss of calcium with the process of moulting. We corroborate this discussion by specifically deducing the crucial role of calcium in the form of ACC and other inorganic components such as phosphates for the expression of inducible defenses in *D.* *magna*.

In conclusion, our study for the first time reveals a detailed description of the chemistry of the carapace of the crustacean *D.* *magna* and the chemical changes occurring in their carapace in association with the expression of inducible morphological defenses against *T.* *cancriformis*. The chemistry of the carapace of *D.* *magna* was characterized using micro-Raman spectroscopic analysis which visualized the presence of a combination of organic and inorganic components constituting mainly α-chitin, proteins and biominerals like amorphous calcium carbonate (ACC) and phosphates (may be inclusive of amorphous calcium phosphate (ACP)). While biominerals such as ACC have been previously found in the exoskeleton of malacostracan crustaceans, e.g. lobster*s* ^3,4^, crabs ^3^ and crayfish ^6^*,* it has previously not been shown to be present in entomostracans. Our study is therefore the first to reveal that also entomostracans, like *D.* *magna*, utilize ACC as a biomineral phase in their exoskeleton. Our results indicate that ACC and phosphates are the most significant chemical components in the carapace contributing to the inducible morphological defenses in *D.* *magna* which is in concordance with previous works on the essential role of calcium and phosphates for *Daphnia*^46,47,55^. The increased amount of ACC in the carapace of *D.* *magna* accompanying the expression of prominent morphological defenses might cause an increased rigidity and better resistance to fractures while also making the carapace less prone to being compressed or crushed by predators. We therefore add another dimension to the previously analyzed^23,30,52^ ultrastructural changes in inducible carapace defense formation of *Daphnia*. Exploring the chemical composition and the modifications of the exoskeleton of this ecologically and environmentally important model organism could be crucial to estimate the impact of environmental conditions, e.g. water chemistry, which may have an influence in predator prey interactions.

## Methods

### Induction experiment

A laboratory cultured clone of *D.* *magna* (clone: K_3_4J) originating from a fishpond in Munich was used. As predator, a laboratory cultured clonal line of *T.* *cancriformis*, originally provided by Erich Eder (University of Vienna), was used. The experiment was conducted in a climate chamber (20 °C + 1 °C) with a day-and-night-simulating photoperiod (15 h light; 9 h darkness). For the experiment, artificial M4 medium ^56^ was used. 2L- glass beakers filled with 2L medium were used as experimental vessels. The water surface of each beaker was powdered with a pinch of cetylalcohol (Tokyo Chemical Industry Co., Nihonbashihonchon, Japan) to reduce surface tension. The beakers were each equipped with a mesh-cage for the predator made of acrylic glass and gauze (mesh width: 180 µm). Before the start of the experiment a pre-induction was performed to ensure a stronger expression of the defense. A total of 10 age-synchronized, primiparous (mature) *D.* *magna* were placed in each beaker (10 per treatment), which either contained a meshed cage with *Triops* or without (Control). Predators were fed daily with 15 individuals of *D.* *magna* and daphnids were fed with 1 mg C/L^−1^ of the green alga *Acutodesmus* *obliquus.* The third clutch of the age-synchronized *D.* *magna* was used for the predator-induction experiment. When released, 15 randomly chosen neonate *D.* *magna* were placed in each 2L beaker. The two treatments referred to as “Control” and “*Triops*” were each replicated six times and the overall experiment was conducted analogous to the pre-induction procedure. The mesh-cages were cleaned every other day to remove biofilm from the gauze and to ensure the exchange of medium and therefore kairomones between the cage and the surrounding medium. Every four days the medium was completely renewed to ensure adequate water quality. When daphnids reached maturity, they were preserved in 80% ethanol. This preservative was chosen since preliminary tests confirmed, that it does not alter the chemical composition of the carapace. Subsequently, morphological parameters (body length, body width and tail spine length) were measured using a digital image-analysis-software (CellSens Dimension v.1.11, Olympus Deutschland GmbH, Hamburg, Germany). The following morphological parameters of all animals were measured, since these are altered in *D.* *magna* in response to *T.* *cancriformis* ^33^: the body length, defined as the distance from the compound eye to the base of the tail spine, body width, defined as the maximal length between the dorsal and ventral edge of the carapace and the tail spine length defined as the distance between the base and the tip of the tail spine (Fig. 3a).

### Sample preparation for Raman-analysis

#### I. Micro-Raman spectroscopy measurements

For the Raman measurements eight preserved *D.* *magna* from each treatment were randomly selected and dissected. Before *Daphnia*-selection, the conserved samples of each replicate per treatment were pooled in an Eppendorf-rack and then the samples were randomly selected. The daphnids were dissected, wherein the helmet, intestines and eggs of the organism were removed using micro-dissecting tools. The empty carapace samples were rinsed in 90% ethanol and placed on a glass slide. The single carapace of each sample was then dehydrated in a desiccator for 24 h and transferred onto a quartz glass slide for the analysis. All Raman measurements were performed using 8 replicates (8 carapace samples of individual *D.* *magna*) per treatment unless otherwise stated.

#### II. Micro-Raman imaging

For the acquisition of Raman images, two randomly selected daphnids of each treatment (one control and one *Triops-*exposed *D.* *magna*) were dissected as described above and the carapace samples were cut next to the dorsal ridge. Then one half of each carapace (of one control and one *Triops*-exposed *D.* *magna*) was placed inside out on a quartz glass slide and cooled on a brass block with crushed ice. After 5 min the carapace was transferred on a warmer glass slide, that had been stored under room temperature, using heat transfer. A glass coverslip was then put over the respective carapace and this was placed in the desiccator for a time span of 24 h prior to Raman image acquisition. One half of each of the carapace per daphnid was used as a replicate for the acquisition of images i.e. one replicate constitutes one individual sample (n = 1). This measurement was repeated using a completely independent sample which is the second replicate to ensure reproducibility of the patterns observed in the Raman images, the results of which are included in the SI.

#### III. Reference spectra

Raman spectra were acquired from reference substances namely crystalline calcium carbonate (Calcite) obtained from Bio-Rad Laboratories GmbH (Munich, Germany), crystalline calcium phosphate (Hydroxyapatite) obtained from Gruessing GmbH (Filsum, Germany) and α-chitin provided by the chair of Biomaterials at the University of Bayreuth.

### Micro-Raman instrumentation

The Raman spectroscopic measurements of the carapace samples were performed on a quartz glass slide using a commercial micro-Raman instrument Witec Alpha RA-300 (Witec, Ulm, Germany). The measurements were performed in a single spectral acquisition mode for the generation of data from specific points across the carapace samples. A large area scanning mode was used for the generation of Raman images of the samples. A frequency doubled Nd-YAG laser with a wavelength of 532 nm was used as an excitation source. The exciting laser radiation was coupled to a Zeiss microscope through a wavelength specific single mode optical fiber. The laser beam was focused onto the sample by means of a 50 × Zeiss objective (NA = 0.7) providing a lateral resolution of ~ 0.5 µm. The focal length of the spectrometer is 800 mm and is equipped with a grating having a groove density of 600 lines per mm to give a spectral resolution of about ~ 3–4 cm^−1^. The laser power used was approximately 15–20 mW at the fiber, depending on the sensitivity of the sample to laser radiation. The Raman scattered light was detected by a Peltier cooled CMOS (complementary metal oxide semiconductor) based CCD detector with a resolution of 1600 × 200 pixels. The Witec Raman microscope has a true confocal design and allows to achieve a depth resolution of ~ 2 µm with the use of a 50 × objective and 532 nm wavelength laser. The instrument was operated by the Witec instrument integrated Control Five software version 5. All the spectra from the samples were acquired in the spectral range between 3600–500 cm^−1^. Additionally, Raman images were acquired by executing a large area scan based on point by point spectral mapping method with a step size between ~ 400–500 nm covering an approximate range of 100 × 100 µm in x and y directions. The Raman images required an acquisition time of ~ 16–18 h and comprised of a data set constituting a total of 150 × 150 spectra per acquired image.

### Raman data analysis

All Raman spectra were pre-processed (baseline correction, vector normalization) and statistical analysis are carried out using GNU R version (2.15.3 and 3.5.1)^57^ and OriginLab software version 8.5 was used for plotting of data. All Raman spectra are an average of ~ 200 spectra acquired from 8 different replicates (8 carapace samples of *D.* *magna*) of the two treatments (Treatments: Control; *Triops*) unless otherwise stated. The Raman images were processed using the Witec integrated Project software (Witec Project version 5.1). The color codes in all the Raman images are based on the standard deviation of integrated normalized Raman intensities corresponding to specific spectral signatures. Furthermore, the Raman images were analyzed by true component analysis (TCA) coupled with spectral demixing using the Witec Project software (version 5.1) to visualize the spatial distribution of different components within the Raman image (example; organic and inorganic components). TCA creates intensity distribution images that show the distribution of different components and creates an average of each component and shows their respective percentages within the Raman image. Spectral Demixing is an algorithm that allows to subtract several single spectra from each other in order to create pure component spectra from mixed spectra.

In addition, a classification model was built by using linear discriminant analysis (LDA) for the classification of the preprocessed spectral data corresponding to control group and the *Triops*-exposed group. Firstly, a principal component analysis (PCA) is performed to reduce the dimension of the data and avoid overfitting of the model. PCA-LDA is a multivariate analysis method that uses a set of principal components (PCs) which best describe the variances present within a group of data ^58,59^. The LDA is then used to project the variances between different groups of data by finding projections that maximizes the variances between groups. The PCs that describe the largest variances within the data can be used as inputs for performing LDA which projects data into a vector space wherein classification information between groups can be efficiently extracted. In our study, the first 22 PC's which allowed for establishing ~ 76% of data variance for Raman spectra of control and *Triops*-exposed *D.* *magna* were used to build the LDA classification model. LDA classification model was built using a total of 400 spectra, 200 spectra from the control group and 200 spectra from the *Triops*-exposed group. Raman spectral data are acquired from 8 replicates (8 carapace samples of *D.* *magna* from the control and *Triops*-exposed treatment respectively) for each of the group (25 spectra per sample per replicate in each group).

### Scanning electron microscopy (SEM)

To get an overview of the structure of the carapace of *D.* *magna*, scanning electron microscopy (SEM) was used. For analysis via SEM a total of four randomly picked *D.* *magna* of the control treatment were dissected as described above (Sample preparation for Raman-Analysis, I.) but not stored after dissection. They were further processed following a modified procedure described previously by Laforsch and Tollrian ^60^ with an dehydration procedure using ethanol and then treated with HMDS (1,1,1,3,3,3-Hexamethyldisilazane) and then dried in a desiccator for 24 h. The dried carapace were mounted on pin stubs (Agar Scientific Ltd., Stansted, Essex, Great Britain) with a conductive adhesion graphite-pad (PLANO GmbH, Wetzlar, Germany) and then fractured with micro dissecting tools. The samples were examined with a Zeiss Ultra plus (FE-SEM with Schottky-field-emission cathode; in-lens detector, SE2 detector, EsB, AsB, EDS UltraDry SDD Thermo Fisher Noran) using an accelerating voltage of 3 kV. The samples were vapor coated with carbon (using a Leica EM ACE600) prior to SEM imaging.

### Statistical analysis

To verify the expression of inducible morphological defenses in the induction experiment, the recorded morphological parameters were compared between treatments. Before statistical analysis, relative values for body width and tail spine length were calculated to compensate for size dependent differences. This was done by dividing the respective trait length through body length. The relative values were then arc-sin-square root-transformed to accomplish the requirements for analysis using a t-test ^61^. Then the replicate-means for each trait were calculated. The means thereby obtained were tested for normality using the Shapiro–Wilk-test and for homogeneity of variances using Levene’s-test and were then compared using a t-test. All graphs and statistics were calculated and plotted with the software package IBM SPSS v.21 (IBM SPSS Statistics Version 21, 64-Bit Edition, IBM Deutschland GmbH, Ehningen, Germany) and R Studio respectively.

## Supplementary Information

Supplementary Figure S1.
