# Supplementary material for: Uncovering the chemistry behind inducible morphological defences in the crustacean Daphniamagna via micro-Raman spectroscopy
Source: Sci Rep. 2020 Dec 29;10:22408. doi: 10.1038/s41598-020-79755-4 (PMC7772340; doi:10.1038/s41598-020-79755-4)
Supplement: Supplementary file 1 — Supplementary Figure S1. [file 41598_2020_79755_MOESM1_ESM.pdf]

Uncovering the chemistry behind inducible morphological defences in the crustacean *Daphnia magna* via micro-Raman spectroscopy

Sven Ritschar[1], Vinay Kumar B. N[1], Max Rabus[1] and Christian Laforsch[1, *]

[1]Department of Animal Ecology I, University of Bayreuth, Germany

*Corresponding author:

Christian Laforsch

Christian.Lafosch@uni-bayreuth.de

OVERVIEW

Supporting information consists of figures supporting the results and discussion parts of the main text.

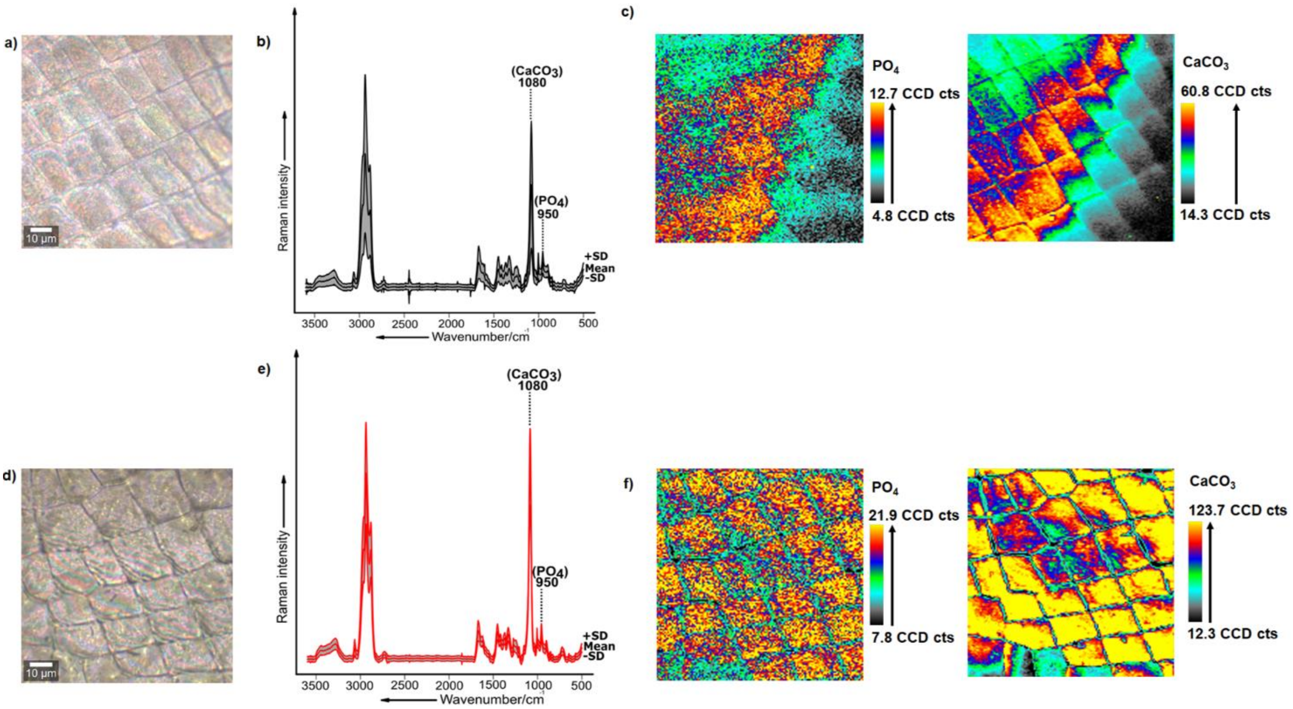

Figure S1. Distribution of ACC and phosphates within the control carapace and *Triops*-exposed carapace of *D. magna* (**second replicate sample**) monitored via micro-Raman spectroscopic imaging; Upper panel, a) Bright-field microscopic image of the control carapace sample b) Mean Raman spectrum ±1 standard deviations of the respective carapace sample (calculated from n=22500 Raman spectra) with regions highlighting the Raman bands corresponding to phosphates (950 cm^{-1}) and ACC (1080 cm^{-1}) respectively c) False colored Raman images acquired from the control carapace sample (n=1) indicating the differential intensity distribution of the Raman bands corresponding to phosphates (left) and ACC (right) respectively. Lower panel, d) Bright light microscopic image of the *Triops*-exposed carapace sample, e) Mean Raman spectrum ±1 standard deviations (SD) of the respective carapace sample (calculated from n=22500 Raman spectra) with regions highlighting the Raman bands corresponding to phosphates (950 cm^{-1}) and ACC (1080 cm^{-1}), respectively, f) False colored Raman images acquired from the *Triops*-exposed carapace sample (n=1) indicating the differential intensity distribution of the Raman bands corresponding to phosphates (left) and ACC (right), respectively. CCD counts (CCD cts.) on the color scale represents relative intensity variation of Raman signatures corresponding to phosphates and ACC within the Raman image.
